# IUC26698-96 Durvalumab + BCG therapy in BCG-naïve, high-risk NMIBC: efficacy and safety analyses from POTOMAC

**DOI:** 10.1093/oncolo/oyag286.009

**Published:** 2026-08-21

**Authors:** S Hussain, N Shore, H Nishiyama, J Palou Redorta, M Krawczynski, A Seyitkuliev, F Guerrero-Ramos, M Kato, T Powles, M De Santis

**Affiliations:** Sheffield - United Kingdom; Carolina Urologic Research Center, Mrytle Beach - United States; University of Tsukuba Department of Urology, Tsukaba - Japan; Urology Department at Fundació Puigvert, Barcelona - Spain; KRAWCZYNSKI MEDICAL CENTER, Lodz - Poland; St. Petersburg Research Center of the Russian Academy of Sciences, St.Petersburg - Russian Federation; Hospital Universitario 12 de Octubre, Madrid - Spain; Osaka Metropolitan University Graduate School of Medicine, Osaka - Japan; Barts Cancer Institute, London - United Kingdom; Charité - Universitätsmedizin Berlin, Berlin - Germany

## Abstract

**Background:**

Durvalumab (D) plus BCG induction (I) + maintenance (M) vs BCG (I+M) demonstrated statistically significant, clinically meaningful improvement in disease-free survival (DFS) in patients with BCG-naïve, high-risk non-muscle-invasive bladder cancer (NMIBC; POTOMAC, NCT03528694). We report in-depth efficacy and safety analyses for D+BCG (I+M) vs BCG (I+M).

**Methods:**

Patients were randomized 1:1:1 D+BCG (I+M), D+BCG (I), or BCG (I+M). For D+BCG (I+M) vs BCG (I+M), we report time to cystectomy (TTC; secondary endpoint), post-hoc exploratory analyses of time to high-risk event, BCG-unresponsive disease, median TTC, cystectomy-free survival (CFS; time from randomization to cystectomy/death); and outcomes in patients with papillary tumours.

**Results:**

Fewer high-risk disease events with D+BCG (I+M) (53/339) vs BCG (I+M) (69/340). Median time to high-risk event was 14.1 vs 8.3mo; 45% (24/53) vs 61% (42/69) patients had early high-risk events (≤1 year from randomization). At recurrence, 65% (24/37) vs 81% (44/54) met BCG-unresponsive criteria, respectively. TTC had HR 0.63 (95% CI 0.31–1.24) with D+BCG (I+M) vs BCG (I+M), medians not reached in either arm (ITT population); among patients who underwent cystectomy, median TTC was 19.0 vs 14.1mo. CFS favoured D+BCG (I+M) (HR 0.69; 95% CI 0.48–0.99). Efficacy in papillary subgroups is summarised (Table), and safety was consistent with the overall population.

**Conclusions:**

Fewer early high-risk events occurred with D+BCG (I+M) vs BCG (I+M), with delayed time to high-risk events, and fewer BCG-unresponsive recurrences. TTC and CFS favoured D+BCG (I+M) vs BCG (I+M). DFS benefit observed across papillary subgroups with D+BCG (I+M).

**Funding:**

AstraZeneca.

**UC26698-96 Table I:** 

D+BCG (I+M) vs BCG (I+M)	DFS		Overall survival	
	HR (95% CI)	Reduction in risk of disease recurrence, progression or death	HR (95% CI)	Reduction in risk of death
**Any papillary tumors (91% of ITT) **	0.61 (0.43–0.85)	39%	0.76 (0.49–1.16)	24%
**Papillary only (64 − 65% of ITT) **	0.56 (0.37–0.84)	44%	0.68 (0.42–1.09)	32%
**Any T1 (58 − 62% of ITT) **	0.55 (0.36–0.83)	45%	0.67 (0.39–1.14)	33%
**T1 only (40 − 43% of ITT) **	0.48 (0.28–0.79)	52%	0.52 (0.27–0.93)	48%
**T1 high grade/grade 3 only (30 − 34% of ITT) **	0.55 (0.31–0.94)	45%	0.58 (0.29–1.12)	42%

HR < 1 favours D+BCG (I+M) vs BCG (I+M).

